# Therapeutic Drug Monitoring of Sputum Voriconazole in Pulmonary Aspergillosis

**DOI:** 10.3390/pharmaceutics14081598

**Published:** 2022-07-30

**Authors:** Sacha Sarfati, Julien Wils, Timothée Lambert, Céline Mory, Laurent Imbert, Gilles Gargala, Hélène Morisse-Pradier, Fabien Lamoureux

**Affiliations:** 1 UNIROUEN, INSERM U1096, CHU Rouen, Department of Pharmacology, Normandie University, F-76000 Rouen, France; sacha.sarfati@chu-rouen.fr (S.S.); julien.wils@chu-rouen.fr (J.W.); cmory@ghpsj.fr (C.M.); laurent.imbert@chu-rouen.fr (L.I.); 2 Department of Pneumology, CHU Rouen, F-76000 Rouen, France; tlambert@ch-alpes-leman.fr (T.L.); helene.morisse@chu-rouen.fr (H.M.-P.); 3 Laboratory of Parasitology-Mycology, EA7510 Rouen University Hospital, F-76000 Rouen, France; gilles.gargala@univ-rouen.fr

**Keywords:** therapeutic drug monitoring, voriconazole, sputum, trough concentration, pulmonary aspergillosis

## Abstract

Voriconazole is one of the most used antifungal azoles against pulmonary aspergillosis. Therapeutic drug monitoring (TDM) of the voriconazole concentration in plasma is recommended in clinical practice guidelines to prevent treatment failure and toxicity. The aim of this study was to evaluate the feasibility and utility of TDM of the voriconazole concentration in the sputum of patients treated for pulmonary aspergillosis. Fifty sputum and 31 plasma samples were analysed with high-performance tandem mass spectrometry (HPLC-MS/MS) in 24 patients included in the study. The voriconazole concentration was simultaneously assessed in the plasma and sputum in 22 samples. The correlation between the sputum and plasma levels was estimated with a univariate linear regression model, and the observed R^2^ was 0.86. We determined the following equation, C_sputum_ = 0.45 (C_plasma_) + 0.21, which could predict the voriconazole concentration in plasma from sputum. TDM of the voriconazole concentration in sputum is an easy, non-invasive and accurate method with which to evaluate voriconazole exposure in patients with pulmonary aspergillosis.

## 1. Introduction

Voriconazole is one of the most used antifungal therapies to treat pulmonary aspergillosis (PA) as first-line therapy and often needs to be maintained for weeks [1]. With an increasing incidence due to the widespread use of chemotherapeutic and immunosuppressive agents, as well as the high prevalence of chronic lung diseases and better diagnosis of different forms, pulmonary aspergillosis involves more and more out-of-hospital patients [2].

The therapeutic range of residual plasma voriconazole is 1.0–5.5 mg/L to prevent both treatment failure and toxicity, mostly hepatic and neurologic [1,3]. However, the intra-individual variability of pharmacokinetics is high, even with weight-adjusted dosing, and can lead to out-of-range voriconazole trough concentrations [4]. Inflammation may play a key role in intra-individual variability [5] as well as pharmacogenomics [6].

Voriconazole is metabolised by the CYP2C19 cytochrome [7]. Consequently, drug-drug interactions (with the proton-pump inhibitor (PPI)), affecting CYP2C19 activity and *CYP2C19* gene polymorphisms, leading to a poor metaboliser phenotype, are associated with voriconazole overexposure (higher than 5.5 mg/L) [1,4,7]. More recently, it has been shown that a genetic variant of CYP3A4 may alter voriconazole exposure [8]. Therefore, according to clinical practice guidelines, dosing adjustment is recommended based on therapeutic drug monitoring (TDM) [1]. TDM is usually performed by assessment of the plasma concentration before the next dose administration (trough concentration (C_min_)) and two hours after dose administration (for oral therapy) at a maximum concentration (peak concentration (C_max_)) [9].

Voriconazole is known to be distributed in most human tissue and especially in broncho-pulmonary tissue [10,11]. In a steady state, the voriconazole concentration in bronchial mucus is stable and can be measured in the sputum independently of the voriconazole administration time [12]. In healthy volunteers, the voriconazole concentration has been investigated in epithelial lining fluid (ELF) with a close relationship between trough plasma and ELF concentrations, and a higher voriconazole concentration in ELF than in plasma [13]. Similar results have been found in lung transplant recipients, showing again a strong positive linear relationship between trough plasma and ELF voriconazole concentrations [14,15].

To our knowledge, determination of the voriconazole concentration in the sputum for TDM has never been investigated until now, though it may present advantages for patients (non-invasive method), physicians and pharmacologists (i.e., measurement of drug concentration directly at the site of action). In this study, we decided to measure, in a steady state, the voriconazole concentration in the sputum of patients treated with oral therapy for pulmonary aspergillosis, to evaluate the variability of the sputum concentration over time and its correlation with the plasma concentration.

## 2. Materials and Methods

We performed a prospective single-centre observational study at Rouen University Hospital (Normandy, France). The study was approved by the hospital review board on 10 May 2022. Patients were prospectively included between December 2017 and February 2020. All adult patients receiving voriconazole in the Respiratory Department’s wards and day clinic for treatment of pulmonary aspergillosis were included during the study period. Exclusion criteria were the withdrawal of informed consent. Liver and kidney toxicity were assessed in all patients according to best practice guidelines with measurement of liver enzymes and serum creatinine before and after treatment initiation. Standard weight-adjusted doses of voriconazole were used, with 12 mg/kg q12h on day 1, then 6 mg/kg q12h as the maintenance treatment.

The voriconazole concentration was measured in the sputum of patients who had been treated for PA for at least 5 days (steady state of voriconazole pharmacokinetics) when sputum was required for bacteriological testing. Microscopic examination validated the quality of the sputum (<10 epithelial cells, >25 polynuclei per field) according to Bartlett’s criteria [16]. The moriconazole concentration was also assessed in the plasma at the same time if required for standard care.

The measurement of voriconazole in all samples was performed at the Laboratory of Pharmacology and Toxicology (Department of Pharmacology, Rouen University Hospital) using high-performance liquid chromatography (Shimadzu^®^, Kyoto, Japan) coupled with tandem mass spectrometry (3200 Qtrap Sciex^®^, Framingham, Massachusetts, United States) (HPLC-MS/MS), as previously published [7]. After collection, samples were transported to the laboratory where blood was centrifuged for plasma extraction. Samples were usually analysed on the same day, but when this was not possible, plasma and sputum were stored at −80 °C overnight and analysed the next day. Sample preparation included protein precipitation by mixing 100 µL of plasma/sputum with 200 µL of reagent containing a structural analogue of voriconazole as an internal standard (voriconazole-d5 1 µg/mL in acetonitrile). For sputum samples, in case of high viscosity, 10-fold dilution in 0.9% NaCl was performed before mixing; then, 1 µL of the deproteinised supernatant was directly injected into the chromatographic system. LC-integrated online sample clean-up was performed using a perfusion column (POROS R2/20, 2.1 × 30 mm; Applied Biosystems, Waltham, MA, USA) with a loading phase composed of 15 mM ammonium acetate in water. Chromatographic separation was achieved on an Alltima^TM^ HP C18 HL (3 µm, 50 × 2.1 mm; Alltech, Grace Discovery Sciences, Columbia, MD, USA) at 60 °C, and the mobile phase consisted of a mixture of methanol/ammonium acetate 10 mM buffer + 0.1% acetic acid (97/3, *v*/*v*, respectively) at an isocratic flow rate of 0.2 mL/min. Following optimisation of the MS/MS system parameters, voriconazole detection and quantification were performed in the multiple-reaction-monitoring mode (MRM) using protonated [M + H]^+^ voriconazole and voriconazole-d5 as precursor ions (*m*/*z* 350 and 355, respectively). The ion transitions monitored were *m*/*z* 350.0 → 126.9 and *m*/*z* 350.0 → 281.0 for quantitation and confirmation of VCZ, respectively. The method was validated for clinical practice according to European Medicines Agency, US Food and Drug Administration, and ISO15189 guidelines [17,18] and met all the required quality criteria, including selectivity, between- and within-assay accuracy and precision, linearity, upper/lower limits of quantification assessment, matrix effect evaluation and stability. The lower limit of quantification of the assay was 0.1 µg/mL and the upper limit of quantification was 10 µg/mL. A range was established using plasma and sputum from patients not treated with voriconazole.

Statistical analysis was performed using GraphPad (Prism v9) or R software v3.4.1, and the ggplot2 package was used for graphic representations. Quantitative variables were compared with a *t*-test and qualitative variables with a Chi^2^ test or Fisher’s exact test. The correlation between plasma concentration and sputum concentration was tested using a univariate linear regression model with the plasma concentration as the unique predictor.

## 3. Results

**Figure 1:**
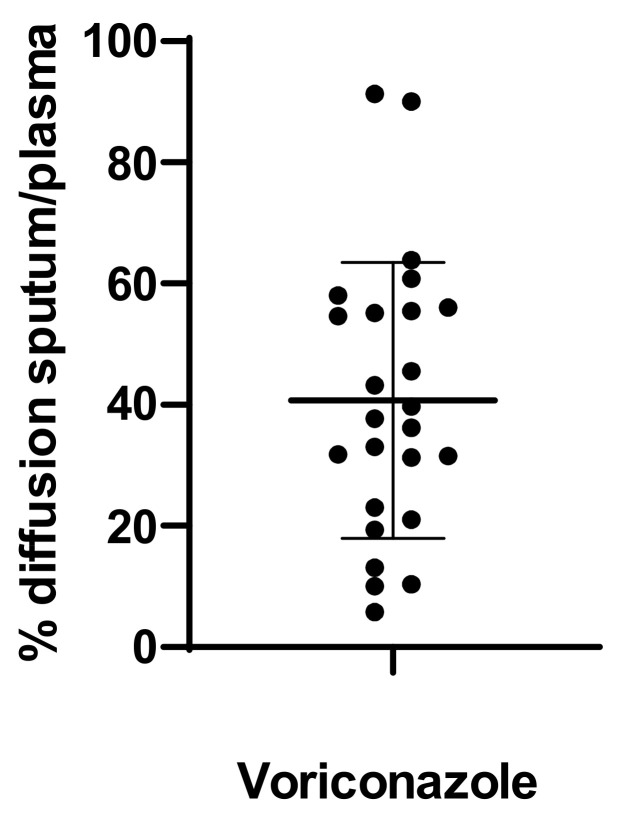
Percentage of voriconazole diffusion rate between sputum and plasma.

**Figure 2:**
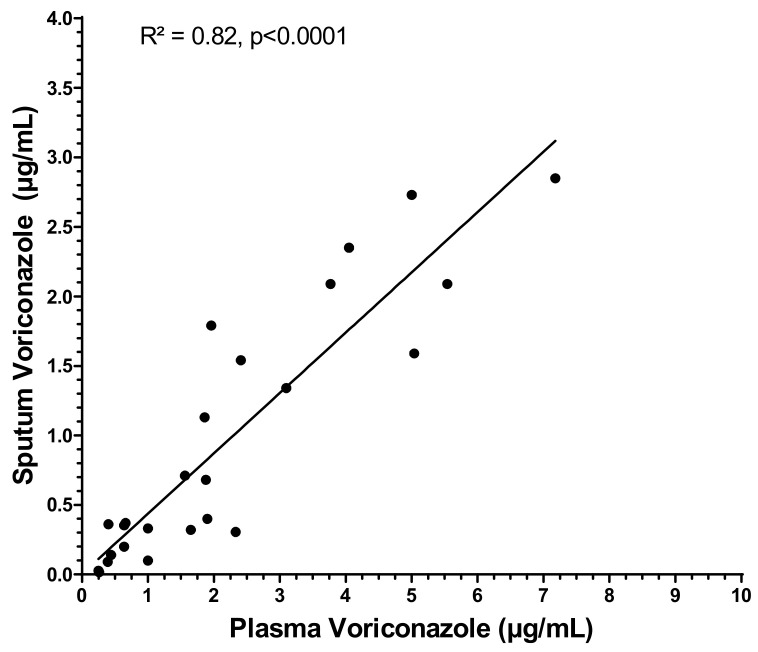
Correlation between plasma and sputum voriconazole concentrations.

Patient characteristics are presented in Table 1. Twenty-four patients were included in the study during 30 TDM sequences (some patients were tested twice several weeks apart) from which 50 sputum and 31 plasma samples were collected. Voriconazole was always detected in the sputum. In 22 cases, plasma and sputum samples were collected simultaneously. The ratio between the plasma concentration and sputum concentration was 0.40 ± 0.22 (Figure 1). A significant correlation between the plasma concentration and sputum concentration was observed (R^2^ = 0.86, *p* <0.01). This correlation can be described with the equation C_plasma_ = (C_sputum_ − 0.21)/0.45 (Figure 2).

**Figure 3:**
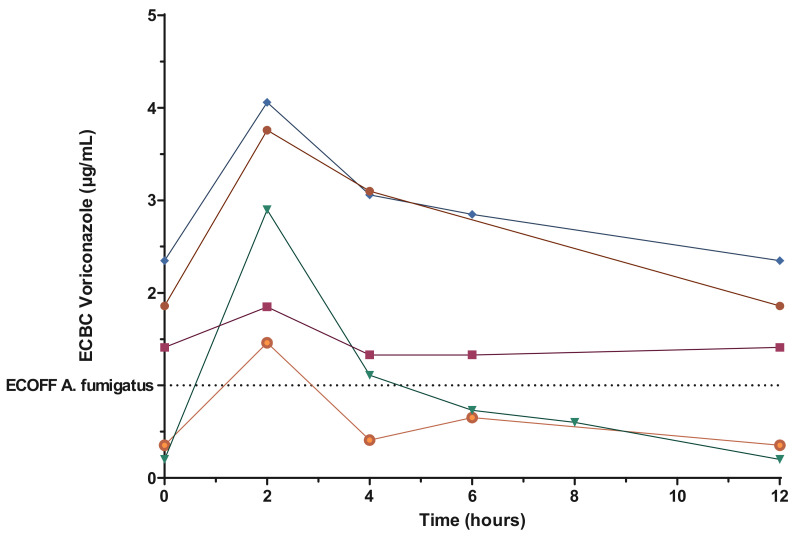
Concentration of voriconazole in sputum over time after dose administration for five patients.

In five patients, the concentration of voriconazole in the sputum was measured over time, with sputum collection during a dose-dose interval at H0 (before dose administration), H2, H4, H6 and H12 (before next dose). These sputum kinetics of the drug showed a peak occurring around 2 h after dose administration, similar to the plasma, but possibly with a smaller amplitude (Figure 3).

**Figure 4:**
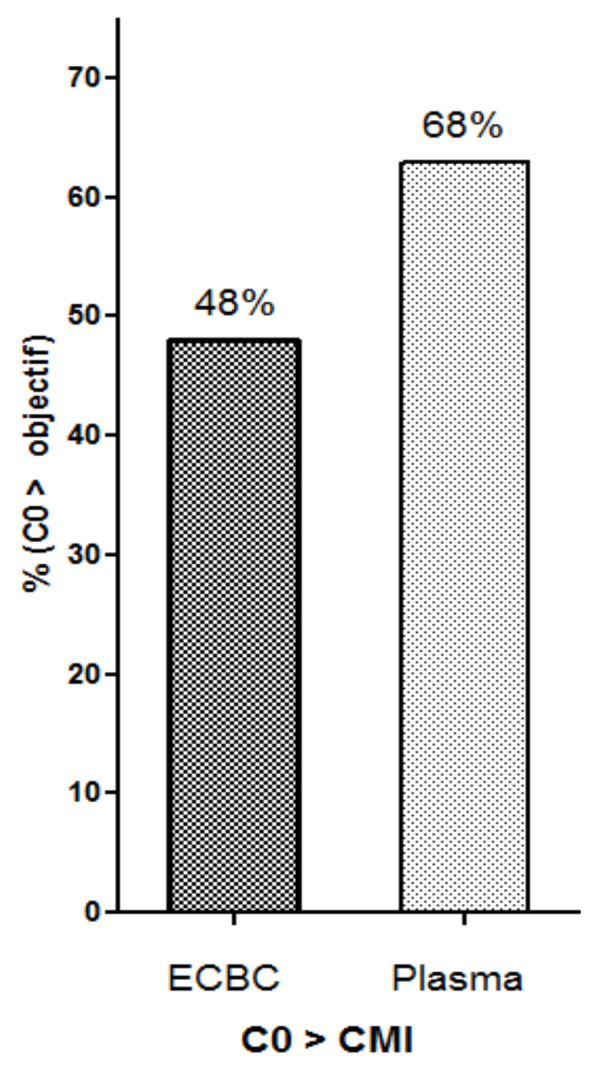
Percentage of samples with Cmin > MIC in sputum and plasma. MIC = minimum inhibitory concentration.

When available, the sputum concentration was compared to the minimum inhibitory concentration (MIC) of *Aspergillus* spp. or the epidemiological cut-off (ECOFF) value of 1 mg/L according to ESCMID guidelines [19]. All isolates were voriconazole-susceptible. Sputum C_min_ was higher than MIC in 48% of cases compared to 68% in plasma (Figure 4).

## 4. Discussion

TDM of voriconazole in plasma for the treatment of PA is recommended but is invasive for the patient and has to be done at a specified time (before daily administration). In this study, we have shown the feasibility of performing TDM of voriconazole in the sputum. Only a small sample of sputum was necessary (>1 mL). Voriconazole was present in all sputum samples, confirming the good penetration of the drug in pulmonary tissues and mucus. To our knowledge, this is the first study to show the results of azole antifungal measures in sputum. We have shown that, in a steady state, the sputum concentration of voriconazole is stable over time, with an absorption peak lower than in plasma. This allows us to perform TDM in sputum independently of the time of drug administration, which facilitates its use.

Moreover, measurement in the sputum may be more relevant as it may more accurately reflect the concentration at the infection site. As such, a plasma concentration higher than MIC is not always an indicator of sufficient pulmonary exposure. A sputum concentration higher than MIC may be considered a better achievement of the pharmacokinetic target. Previous studies, designed to investigate the penetration of voriconazole in the lungs, showed opposite results regarding the correlation between plasma and lung concentrations, with higher levels in the latter [13,14,15]. Several hypotheses may explain this difference. First, these studies were based on the measured concentration in ELF, which is a different fluid of the lung. Sputum contains, in addition to ELF, bronchial and tracheal mucus and cellular remains, in which the penetration of voriconazole may be different. Plus, the concentration in ELF is established from bronchoalveolar lavage (BAL), which involves injecting the lung with a significant amount of saline, then the measured concentration of the drug is corrected with the ratio of urea concentration in the BAL compared to the plasma. This method is more likely to present a calculation bias compared to the direct measurement of voriconazole in the sputum, as in our study. Finally, previous studies were performed in non-infected lungs. The local inflammation caused by PA is likely to alter the penetration of the drug, which is one of the reasons why TDM of voriconazole in the sputum may be interesting in clinical practice.

The main limitation of this method was the lack of availability of sputum. However, respiratory disorders usually increase the productivity of coughing and patients are trained to spit regularly for bacteriological testing. In our study, sputum samples were collected during induced coughing by a respiratory physiotherapist. This technique is routinely used in respiratory medicine departments, but still requires training to ensure the good quality of the samples. This training aspect may limit the use of this method as TDM in other settings than respiratory wards or day-clinics.

Another limitation of our study was that we used Bartlett’s criteria to ensure minimal saliva contamination of the sample, which are designed for bacteriological and not pharmacological testing. In the absence of better criteria for pharmacological analysis of sputum, we cannot rule out a role of saliva contamination in affecting the concentration variability. Although the size of our population was quite small, all our patients were in a steady state of treatment for PA with a standard dosing regimen, which ensured the good external validity of our method. However, analyses involving more patients from multiple centres will be needed to confirm our results in the future.

## 5. Conclusions

In this article, we described an innovative method for the TDM of azole antifungals in outpatients treated for PA. The feasibility was easily demonstrated, as it does not require a different measurement method than in plasma. The value of TDM in sputum compared to plasma still needs to be assessed in larger studies. However, we propose that it may more accurately reflect the exposure of the infection site and it can be performed more easily and independently of the voriconazole administration time. This innovative method of measuring the drug concentration in the sputum has shown promising results for the overall benefit of patients with pulmonary aspergillosis.

## Figures and Tables

**Table 1:** Patient characteristics.

Characteristic	Value
Age (mean ± SD)	54 ± 14 years
Sex, male (*n* (%))	13 (54)
Weight (mean ± SD)	73 ± 13 kg
Height (mean ± SD)	170 ± 8 cm
BMI (mean ± SD)	21 ± 3.7 kg/m^2^
Positive *Aspergillus* spp. culture (*n* (%))	16 (66.6%)
Species (*n* (%))	
*Aspergillus fumigatus*	14 (87.5%)
*Aspergillus neoellipticus*	1 (6.25%)
*Aspergillus wellvitchiae*	1 (6.25%)
Voriconazole daily dose	436 ± 98 mg

## Data Availability

The data presented in this study are available on request from the corresponding author.
